# Marine synthetic ecology: From microbial communities to ecosystems

**DOI:** 10.1016/j.isci.2026.114704

**Published:** 2026-01-15

**Authors:** Ruilin Su, Xiaoli Yu, Mingyang Niu, Kun Wu, Hongbin Liu, Qingyun Yan, Zhili He

**Affiliations:** 1 Marine Synthetic Ecology Research Center, Southern Marine Science and Engineering Guangdong Laboratory (Zhuhai), Zhuhai 519082, China; 2 Department of Ocean Science, Hong Kong University of Science and Technology, Hong Kong 999077, China

**Keywords:** environmental science, ecology, microbiology

## Abstract

Marine ecosystems are vital for global ecological functions and human health but face escalating threats from both anthropogenic activities and global climate change. Marine microorganisms, comprising over two-thirds of the marine biomass, play essential roles in biogeochemical cycling of elements and food webs. Synthetic ecology aims to understand ecological theories and provide nature-based solutions to emerging ecological and environmental problems. Here, we first propose a framework, the resource-design-verification-evolution-application (RDVEA) model for marine synthetic ecology studies, aiming to understand marine ecological theories, design and construct marine-specific synthetic communities, and optimize their functionality toward marine environmental applications. Synthetic ecosystems may target food web interactions, greenhouse gas mitigation, and carbon sequestration enhancement in the future marine environment.

## Graphical abstract

**Figure:**
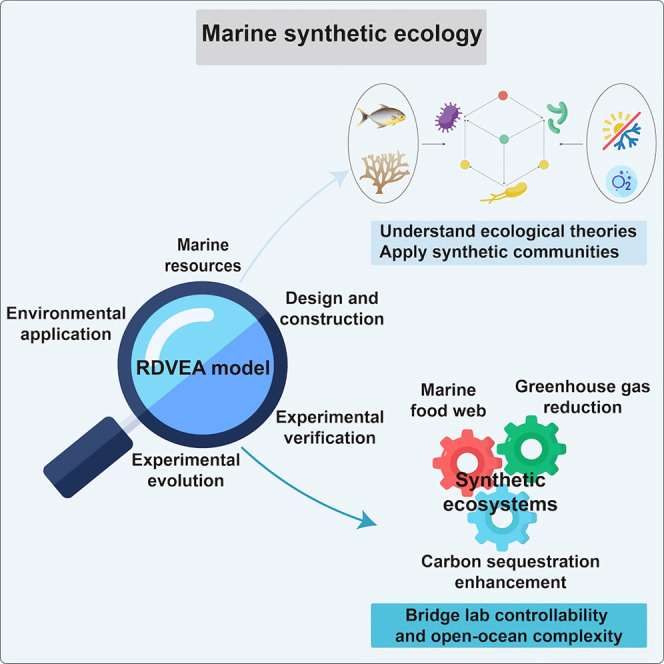

## Introduction

Marine environments cover approximately 71% of the Earth’s surface with an average depth of 3.8 km, encompassing diverse ecosystems such as blue carbon (C) ecosystems (e.g., mangroves, saltmarshes, and seagrasses), coral reef ecosystems, and deep sea ecosystems.^1^ These ecosystems are interdependent with the organisms they support, including animals, plants, microorganisms, and viruses, which also interact with each other and play critical roles in maintaining ecological balance and ecosystem functions.^2^ For instance, coral reefs, and blue C ecosystems can purify seawater, protect biodiversity, shield coastal communities from flooding and erosion, and mitigate global change by orchestrating biogeochemical cycles and food webs in marine ecosystems.^3^^,^^4^ Microscopic organisms accounting for over two-thirds of the marine biomass are essential for sustaining such biogeochemical cycles and underpinning food webs in these ecosystems.^5^ However, current anthropogenic activities and global change severely threaten marine ecosystems and speed up their degradation, leading to the frequent occurrence of harmful algal blooms (HABs), plastic pollution, and coral bleaching.^6^ Meanwhile, traditional conservation initiatives (e.g., waste regulation and fishing restriction) are insufficient to tackle these complex challenges.^7^ Also, the complexity of marine communities frequently results in unforeseen high-order phenomena largely due to a lack of comprehensive understanding of ecological theories and novel remediation technologies.^8^ Therefore, novel theories and innovative technologies are urgently needed to lessen the degradation of marine ecosystems with a focus on the biotic and abiotic interactions, especially among microorganisms and their associated animals and plants under environmental and climate change.

Synthetic microbial ecology was recently developed for understanding mechanisms behind microbial interactions, functions, dynamics, and evolution by constructing controllable synthetic microbial communities, opening new avenues for environmental applications.^9^ Synthetic microbial communities could achieve high stability and emergent functions beyond monocultures by harnessing cross-feeding or cooperative interactions (e.g., methanogens exchanging hydrogen with sulfate-reducing bacteria), resource sharing, competition, and parasitism.^10^ The construction of synthetic microbial communities includes a top-down approach, which simplifies existing complex systems through enrichment, and a bottom-up approach, which artificially engineers simple systems from available isolates.^11^ Due to their simplicity and high modularity, these systems enable experimental interventions and mathematical modeling to simulate and verify ecological interactions and underlying mechanisms among prioritized strains. These greatly enhance our understanding of community functions, interactions, dynamics, and evolution of marine microorganisms by establishing causal links between microbiome composition or activity and host phenotypes.^9^^,^^11^ For example, we constructed synthetic nitrifying communities to explore niche differentiation and interaction mechanisms among different types of nitrifiers (e.g., ammonia oxidizing archaea, ammonia oxidizing bacteria, nitrite oxidizing bacteria, and complete ammonia oxidizing bacteria),^12^ and synthetic denitrifying communities to study the relationship between biodiversity and community function and stability through long-term experimental evolution.^13^^,^^14^ Also, synthetic communities could engage in negative interactions with pathogens, serving as both prophylactic and bioremediation strategies to eliminate pathogens and control diseases in plants and animals.^15^ Additionally, three-dimensional (3D) printing could construct embedded synthetic communities with greater resistance to environmental disturbances and toxins, increasing their viability and stability in environmental applications.^9^^,^^16^ These results indicate that synthetic microbial ecology offers great potentials for us to understand biotic and abiotic interactions of microbial communities and provides novel strategies for environmental remediation.

Here, we extend synthetic microbial ecology to marine synthetic ecology, a new interdisciplinary emerging ecology, biology, engineering, and technology to engineer sustainable ecosystems using natural strategies, enabling ecosystems to be strong, adaptable, resilient, and stable. This focus is critical, as marine synthetic ecology must address challenges specific to the marine environment, such as high salinity, immense scale, global connectivity, and globally significant biogeochemical processes such as the biological carbon (C) pump.^17^ Marine synthetic ecology aims to design and construct *de novo* marine biological communities toward better understanding of ecological rules in marine ecosystems, especially biotic and abiotic interactions and underlying mechanisms for enhancing marine ecosystem functioning with highly efficient synthetic communities. On the one hand, such synthetic communities constructed with various organisms (e.g., bacteria, archaea, fungi, plants, animals, and viruses) are used to study their niche differentiation, interaction mechanisms, and their environmental adaptability, forming general ecological principles. On the other hand, synthetic communities with high efficiency, adaptability, and stability can be applied to various marine ecosystems by enhancing their ecosystem functionality like through long-term evolution.^6^ It is expected that marine synthetic ecology will emerge as a vital, interdisciplinary field for enhancing marine ecosystem functions and services, and the construction of synthetic communities and synthetic ecosystems can further improve marine-specific functions, such as HAB control, nutrient cycling balance, pathogenesis control, environmental remediation, and global climate change mitigation. However, the transition from gaining marine resources to environmental application represents a monumental challenge in efficacy, functionality, and stability. This gap presents scientific questions as follows: (1) how can we find ecology principles governing marine community functions, interactions and evolution with synthetic communities? (2) how can we move beyond observations of complex marine communities to actively and predictably apply synthetic communities for enhancing marine ecosystem resilience and functioning? To address these fundamental questions, our core hypotheses include (1) that general ecology theories found in well-studied ecosystems (e.g., soil and water) are able to adopt to marine ecosystems, and be tested by synthetic ecosystems and (2) that synthetic communities can be scaled to marine ecosystems and this leap requires a structured, iterative, and cautious framework from concept to application. To realize these goals, we propose a framework named the resource-design-verification-evolution-application (RDVEA) model as the necessary methodological roadmap.

## The RDVEA model

**Figure 1:**
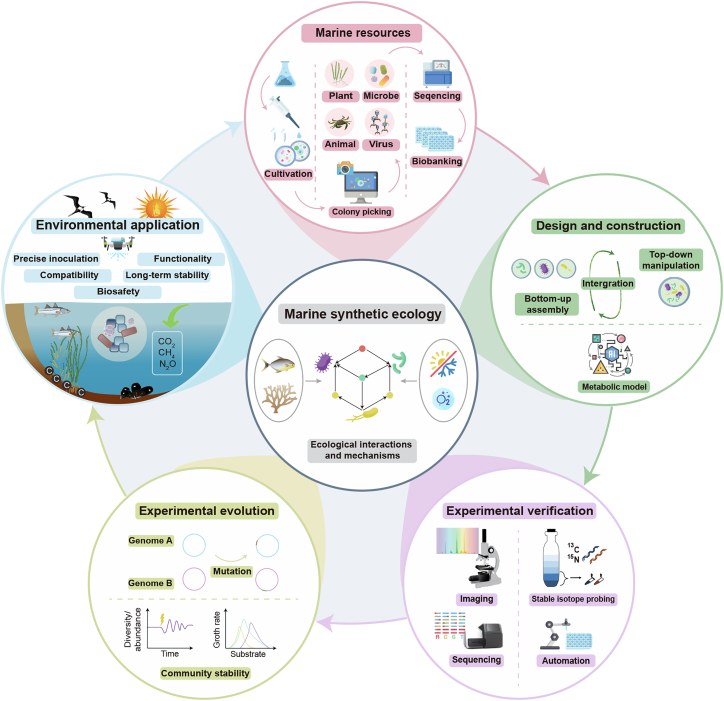
A framework for marine synthetic ecology studies using the resource-design-verification-evolution-application model This RDVEA model comprises five key elements: collection of marine biological resources, design and construction of synthetic communities, experimental verification of functionality, enhancing adaptability and stability through experimental evolution, and environmental applications of synthetic communities. This model aims to advance our understanding of marine ecological principles and enhance ecosystem functioning. Organismal icons are extracted and edited from the University of Maryland Center for Environmental Science Integration and Application Network and Faticon.com. Note that creatures’ sizes are not to scale.

The RDVEA model provides a clear roadmap for marine synthetic ecology studies and environmental applications through five key stages: (1) the enrichment and isolation of key marine organisms to serve as foundational biological parts; (2) the rational design and construction of synthetic communities using predictive models and ecological principles; (3) the experimental verification of synthetic communities’ functions and interactions to advance the understanding of ecological principles; (4) the enhancement of community adaptability and stability through techniques such as long-term experimental evolution; and (5) the deployment of optimized synthetic communities for specific purposes, such as increasing ecosystem functions such as greenhouse gas (GHG) reduction and C sequestration enhancement (Figure 1).

### Marine biological resources

The success of marine synthetic ecology hinges on the isolation, identification, and cultivation of key marine organisms, including animals, plants, microorganisms, and viruses, particularly non-model species inhabiting various marine environments. It is worth mentioning that prioritizing the isolation and access of native marine resources can reduce the risk of introducing invasive species and disrupting ecological balance during environmental applications.^15^^,^^18^ Advanced isolation and identification technologies, such as non-targeted methods (e.g., metagenome sequencing and time-of-flight mass spectrometry), and targeted approaches (e.g., whole genome sequencing, nucleic acid amplification, and hybridization), play a pivotal role in this process.^19^ The effectiveness of identification procedures is closely tied to the advancements in cultural techniques, which have improved standard plate-based methods, and introduced culturomics, coculture, cell-target, and *in situ* cultivation approaches to expand access to diverse marine resources.^6^ For example, culturomics facilitates the isolation of not yet cultivated microorganisms, such as extremophiles from hydrothermal vents or taxa outcompeted by dominant species by profiling specific taxa under various culture conditions.^20^ Another key enabling technology is high-throughput dilution-to-extinction cultivation, which uses carefully designed media in multi-well plates to successfully isolate and grow previously uncultured oligotrophic bacteria, significantly expanding the library of available aquatic microorganisms.^21^ Also, advances in artificial intelligence (AI) and machine learning further enhance large-scale, automated isolation of organisms from diverse marine ecosystems through adaptive picking, accelerating the establishment of comprehensive databases cataloging marine organisms and their associated genomes.^22^^,^^23^ Such databases serve as fundamental marine resources for marine synthetic ecology studies and technology development. Yet, a crucial step remains: the deposition of isolates into public biobanks, which ensures the accessibility, reproducibility, and long-term utility of cultures for subsequent synthetic community construction and analysis.^24^

### Design and construction of synthetic communities

Integrating genome information with modeling mass balances, metabolite fluxes, and ecosystems processes facilitates the design and construction of synthetic communities, which may be realized by bottom-up metabolic modeling or top-down process-based techniques.^22^ These complementary approaches can predict microbial assembly, functions, and interactions across varying levels of complexity.^11^ The important work of predictive design is driven by computational tools such as genome-scale metabolic models (GSMMs), which use an organism’s genetic information to simulate its metabolisms and predict their cross-feeding of metabolites, allowing for the rational design of stable and synergistic communities.^25^^,^^26^ To design synthetic communities with high functionality and stability, some strategies can be used to boost their functionality and stability, such as high metabolic complementarity (e.g., cross-feeding auxotrophs), distributed functioning, functional plasticity, low resource competition,^27^^,^^28^^,^^29^ diversification of biochemical roles, and spatiotemporal control.^30^ For example, functional plasticity enables an adaptive “division of labor,” where community members can dynamically shift metabolic roles, reroute energy pathways, and form defensive barriers to maintain the overall system function when resisting specific environmental stressors. This resilience is often supported by metabolic cross-feeding, where even low-abundance non-degraders provide essential metabolites that boost the performance of the entire community.^29^ Also, the distributed functions among multiple species could enhance functionality, genetic stability, and long-term survival of synthetic communities due to a lesser load on individual species^31^; thus, these constructed communities tend to exhibit greater resilience against environmental variations and invasions by non-native species in the field.^31^ Synthetic denitrifying microbial communities can maintain high denitrification efficiency when exposed to plasticizers and antibiotics by dynamically shifting functional roles, rerouting electron flow, and reconstructing metabolic networks.^29^ Synthetic communities constructed with salt-tolerant, or halophilic stains could aid in maintaining ionic homeostasis in the cytoplasm or Na^+^ efflux, thus increasing the salt tolerance of non-halophytic strains.^7^ Additionally, leveraging the relationship between stress responses, signaling molecules, and microbiome assembly can strengthen microbial communities, benefiting stressed plants and animals.^7^ This connection warrants further investigation of developing stress-resilient marine remediation strategies in response to climate change.^7^ After the successful design of synthetic communities, techniques such as 3D printing and microfluidics can help build the heterogeneous marine environments to study community spatial distributions, community dynamics, holobiont interactions, and quorum sensing mechanisms.^26^ Specifically, microfluidics allows for the creation of precisely controlled micro-environments that mimic natural settings, enabling the observation of microbial behaviors such as growth, motility, and inter-species interactions at the single-cell level. This technology is ideal for testing ecological theories in engineered habitats before scaling up.^26^ Additionally, deep reinforcement learning can identify optimal input patterns to enhance the productivity of synthetic communities by operating them under reasonable control policies,^23^ thus future research can leverage existing machine learning algorithms, particularly from metabolic engineering, to advance deep learning applications in the design and construction of synthetic communities.^23^

### Experimental verification of synthetic communities’ functionality

Using complementary methods enables rigorous verification of the desired functionality of constructed synthetic communities and their systemic interactions with the environment. The growth of synthetic communities and the dynamics of individual strains can be monitored by quantitative polymerase chain reaction (qPCR) or spike-ins analysis^24^; the abundance and transcript of key functional genes can be detected by (meta)genome, (meta)ribosomal, and (meta)transcriptome sequencing analysis^24^; advanced proteomic and metabolomics approaches can directly assess the community functionality^32^; imaging tools (e.g., fluorescence microscopy and confocal microscopy) combined with stable isotope probing can provide high-resolution insights into substrate assimilation and metabolite exchange within a synthetic communities.^22^^,^^33^ These complementary “meta-omics” approaches are a cornerstone of verification, allowing researchers to move beyond simple taxonomic descriptions to a system-level understanding of community functioning.^34^

To further distinguish genetic variations among individuals of the same species, the recently developed modular bacterial tags (MoBacTags) can be used to track nearly identical bacterial commensals within a community.^35^ Similarly, novel RNA barcoding technologies can autonomously record information about gene transfer across a community by writing a barcode directly into ribosomal RNA, allowing for sensitive, culture-independent tracking of microbial interactions.^36^ Automated high-throughput systems can be embraced to enhance the efficiency and standardization for testing those built synthetic communities, yielding rich and replicable datasets for big data analytics and machine learning.^9^ In the future, it is indispensable to revisit and develop new modeling algorithms and techniques to create more practicable bioinformatic tools and feasible *in silico* experiments; advanced experimental approaches (e.g., physiological and ecological characterization, molecular technologies, and image analysis) are equally important to improve and verify computer models.^37^

### Experimental evolution for synthetic communities’ adaptability and stability

Experimental evolution enhances the adaptability and stability of synthetic communities by simulating evolutionary processes under controlled conditions, offering insights into how their behavior and adaptation in natural settings. This process, often called directed evolution, is a powerful “top-down” engineering approach that mimics natural selection by subjecting a community to iterative rounds of variation, selection for a desired function, and amplification, thereby leveraging eco-evolutionary forces to find high-functionality and stable synthetic communities.^38^

For synthetic communities to function as cohesive evolutionary units, they exhibit positive interactions, functional integration, and entrenchment.^28^ Enhancing these traits reduce internal competition, fosters metabolic interdependencies, and ensures robustness of synthetic communities across environments.^28^ For example, a synthetic microbial community comprising *Pseudomonas putida* and *Acinetobacter johnsonii* demonstrated that *A. johnsonii* could maintain its abundance with *P. putida* by stabilizing the coexistence of those two morphotypes after 200-generation co-evolution. *A. johnsonii* influenced the evolutionary trajectory of *P. putida* through mechanisms such as resource cross-feeding and buffering niche competition, underscoring the critical role of interspecies interactions in sustaining microbial diversity.^39^ Synthetic communities could also be engineered to enhance specific functions (e.g., productivity and denitrification) by increased complementarity, which has been demonstrated by synthetic denitrifying communities,^14^ and high phylogenetic diversity further enhanced community functions and stability through evolutionary processes.^13^ Laboratory evolution can develop strains that are resistant to specific phage or phage cocktails by inducing mutations that alter cell wall composition, thereby modifying susceptibility to impede phage binding or replication through targeted deletions.^31^

Synthetic communities evolved under various adverse conditions may also establish intricate metabolic pathways (e.g., phenol and formaldehyde degradation) through increased stochastic processes^40^; thus, it is crucial to investigate how long-term experimental evolution can drive irreversible entrenchment, such as through mutations that lock metabolic interdependencies.^28^ Once they are verified and evolved in the laboratory, these communities can be further validated for their multitrophic interaction mechanisms and applied to the marine environment.

### Environmental applications of synthetic communities

Transiting synthetic communities from laboratory research to field applications faces various challenges, including efficient inoculation and colonization, sustainable functionality, compatibility with native communities, long-term stability, and biosafety, which demand critical considerations and innovative technology development.

#### Precise inoculation and efficient colonization of synthetic communities into marine environments

The first step of environmental applications is to inoculate synthetic communities into their target ecosystems. For example, automated drones or boats, augmented with AI-driven spatial mapping tools can facilitate precision inoculation at a large scale^41^; encapsulating synthetic communities in biochar/chitosan microbeads can shield them from environmental variations while enabling slow release during colonization.^42^

#### Sustainable functionality of synthetic communities in the marine environment

Modular design principles partition synthetic communities into functional submodules (e.g., nutrient cyclers),^43^ ensuring the maintenance of synthetic communities functionality in marine ecosystems. Real-time validation with quantitative phase imaging and 3D fluorescence imaging can track microbial growth and interactions^44^; next-generation sequencing can further characterize microbial diversity, metabolic functions, and natural attenuation processes^7^^,^^37^^,^^38^; machine learning and genomic network analysis synergize with these data to predict interactions and simulate metabolic exchanges, identifying keystone species of native microorganisms.^45^^,^^46^^,^^47^ For instance, metagenomic analysis revealed functional phenolic-degrading synthetic communities optimized from flask to bioreactors and field deployment under transient environmental conditions.^40^

#### Compatibility of synthetic communities with native communities

As complex interactions exist between synthetic communities and native communities, the effect of synthetic communities on native communities should be carefully considered, and the ecosystem level function should be comprehensively assessed. Synthetic communities can be designed using dominant and keystone taxa that are naturally better at colonizing and influencing their environments. Additionally, applying ecological concepts such as the “priority effect”—where a synthetic community is introduced at a specific time and order—can give it a competitive advantage in establishing itself before native microorganisms can mount a full resistance. Moreover, to mitigate resource competition caused by synthetic communities—specifically, their potential to outcompete resident taxa for resources—minimal synthetic communities incorporating cross-feeding strains are recommended.^42^^,^^43^ Multi-genome metabolic modeling can guide the design of such minimal communities by identifying essential compounds for microbial metabolism, leveraging insights into microbe-microbe-plant interactions.^48^ For instance, GSMMs can predict the complex cross-feeding interactions between synthetic communities and native microbes.^26^ Beyond design, high-throughput workflow can assess function and compatibility between synthetic and native communities through phenotypic assays amenable to high-throughput screening.^45^^,^^49^

#### Long-term stability of synthetic communities

Synthetic communities’ stability could be enhanced by overlapping metabolic pathways to buffer against species loss, and by integrating with native taxa to bolster nutrient exchange and habitat colonization.^50^ Long-term stability can be monitored using *meta*-omics and nanosensors,^24^ while non-invasive SEER-FISH (sequential error-robust fluorescence *in situ* hybridization) and optogenetic toolkits provide single-cell resolution of spatial and functional dynamics.^46^^,^^47^ Data from these advanced techniques collectively inform periodic reapplications’ schedules and guide iterative redesigns, such as adjusting strain ratios or nutrient dependencies, to refine synthetic communities efficacy in the environment.^10^^,^^51^

#### Biosafety of synthetic communities

First, as synthetic communities are constructed with natural species instead of genetically modified species, environmental risks are minimal. Also, such biosafety risks can be further reduced by prioritizing synthetic communities composed of pervasive native taxa, which inherently reduce biological risks compared to genetically modified organisms.^52^^,^^53^ Moreover, the risk of microbial invasion can be managed by using the beneficial secondary metabolites produced by the synthetic community, thus avoiding the introduction of a potentially invasive species.^54^ Another strategy is to select for microorganisms that are fast-acting but have a “short legacy,” meaning that they are quickly outcompeted by the native community after they perform their functions, preventing long-term persistence.^54^ Moreover, aligning the timing of applications with the host’s key developmental stages, such as flowering, also ensures that the synthetic community provides benefits when most needed. Additionally, environmental risks can be quantified pre-emptively using EcoGenoRisk, a computational tool that screens genetic similarity and ecological relationship (e.g., competition and mutualism) between synthetic communities and native communities.^55^ Moreover, by incorporating the functionality of a proposed synthetic community into an Earth System Model, the potential long-term and large-scale effects of the synthetic community on marine biogeochemical cycling and food webs can be simulated.^56^

By systematically applying this iterative process, the RDVEA framework is designed to deliver two primary categories of detailed outcomes. On the one hand, it moves marine ecology from field observations to mechanistic understanding of fundamental principles that govern marine ecological functions, interactions, and evolution. On the other hand, it provides robust and nature-based solutions to enhance marine ecosystem functioning in response to marine environmental stresses and global change by engineering synthetic communities and synthetic ecosystems. While synthetic communities offer targeted environmental solutions, scaling controlled laboratory experiments to natural marine environments remains critical. Synthetic ecosystems, as simplified yet functionally representative models, mimic transient environmental conditions (e.g., nutrient gradients and redox fluctuations) to decipher multitrophic interactions and underlying mechanisms in synthetic ecology.^47^ These synthetic ecosystems bridge laboratory controllability with environmental complexity, addressing scalability gaps, and guaranteeing the transition from the laboratory to marine ecosystems.^24^

## Construction of synthetic ecosystems

### Synthetic ecosystems decode multitrophic interaction mechanisms

Synthetic ecosystems serve as “proof-of-concept” studies, assessing the impact of synthetic communities in target ecosystems while maintaining ecological complexity. Synthetic ecosystems include microorganisms (e.g., bacteria, archaea, and protists), macroorganisms (e.g., animals and plants), viruses and their suitable environments, and ecological theories focus on multitrophic interactions of organisms (e.g., food webs), element cycling and coupling mechanisms, and energy flow under varying environmental conditions.^22^^,^^40^ For instance, a recent study investigated how surfactin produced by *Bacillus subtilis* influenced the establishment and interactions within synthetic bacterial communities in synthetic soil-mimicking microcosms. Although neither the wild type nor the surfactin non-producer mutant affected the composition of the synthetic communities over time, both *B. subtilis* and the synthetic community’s metabolomes were altered during co-cultivation, highlighting surfactin’s role in facilitating *B. subtilis* success and altering community metabolomes.^32^^,^^46^

### Engineering principles for synthetic ecosystem design

To effectively apply synthetic communities for environmental deployment, several key steps are essential to construct synthetic ecosystems: (1) it is essential to identify the main environmental factors that influence biological processes relevant to desired functions or applications, and this step should isolate biological processes of interest and the effect of various environmental factors on these biological processes; (2) synthetic ecosystems should be crafted to closely replicate these key environmental factors (e.g., pH, temperature, salinity, dissolved oxygen, and tidal cycle) and emulate marine environments as closely as possible, allow to experimentally manipulate species diversity and composition to optimize ecosystem functions^57^; (3) advanced gnotobiotic systems such as EcoFabs and Ecotrons can be used as key platforms for building and monitoring synthetic ecosystems under precisely controlled conditions^47^; and (4) the use of specially designed packaging materials can enhance synthetic communities tolerance to extreme marine environmental conditions. These materials can be combined with genetic mutations or single-cell encapsulation techniques to further enhance synthetic communities’ stability and protection, ensuring their robust performance under specific conditions.^50^^,^^58^

### Synthetic ecosystems use cases

**Figure 2:**
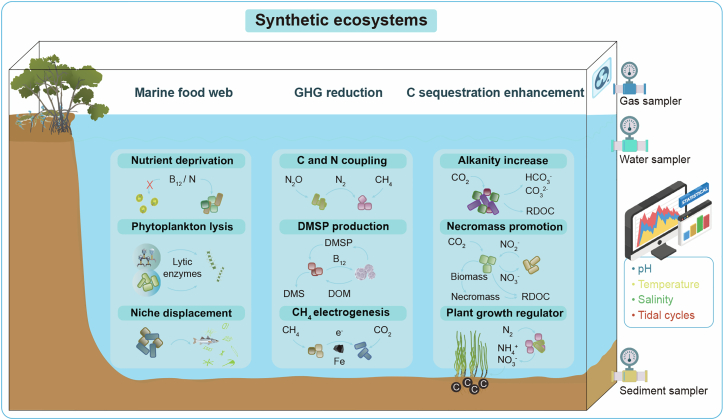
Synthetic ecosystems enable the investigation of food web dynamics, GHG reduction, and C sequestration enhancement N, nitrogen; DMSP, dimethylsulfoniopropionate; DMS, dimethyl sulfide; DOM, dissolved organic matter; RDOC, refractory dissolved organic carbon.

Based on these prerequisites, here we propose three examples of synthetic ecosystems for marine food webs, C sequestration, and GHG mitigation (Figure 2). Beyond their potential as remediation tools, these systems serve as scientific platforms to decipher complex ecological rules, such as coupling mechanisms of biogeochemical cycles, and food web structure and function to ensure that any future applications are grounded in a mechanistic understanding of ecosystem stability rather than efficiency only.

#### Marine food web synthetic ecosystems

These ecosystems represent a frontier research in understanding how multitrophic interactions, including microorganisms, plants, animals, and viruses, mediate ecosystem functioning and stability under environmental perturbations.^59^ These interactions are critical as both human activity- and climate-driven changes (e.g., eutrophication, global warming, and ocean acidification) destabilize food webs. For instance, ocean warming could amplify bacterial activity, and paradoxically bolster food availability for zooplankton, but this could also accelerate viral replication to lyse microorganisms^52^^,^^60^; marine heatwaves could reduce biomass like krill, indirectly starving high trophic level animals (e.g., salmon and seabirds), but also favor zooplankton that outcompete fish for resources.^61^ Such dynamics of food webs underscores the fragility of multitrophic balance, as nutrient-rich coastal environments often trigger phytoplankton proliferation, fostering HABs such as *Alexandrium*, which are auxotrophs for vitamin B_1_ and B_12_.^62^ To address this marine challenge, synthetic ecosystems can exploit the dependency of harmful algae on specific micronutrients by deploying viruses and bacteria that rapidly consume vitamin and nitrogen, which are essential for their growth.^63^ Also, viruses and algicidal bacteria can lyse harmful algae, altering specific harmful algae population abundances.^62^ To ensure the controlled application, these synthetic communities can be immobilized in alginate beads, preventing their release into the marine environment while allowing lytic enzymes to diffuse and exhibit higher algicidal activity comparable to that of their free-living counterparts.^64^ Additionally, melting polar ice and permafrost may release ancient species, which may move into new areas and then displace local species.^65^ Therefore, the projected marine food web synthetic ecosystems will integrate such cross-temporal interactions to simulate how these resurrected “time-travelers” interact with native communities and reconfigure trophic cascades under future climate scenarios. By modeling these dynamics, synthetic ecosystems allow us to verify the mechanisms of multitrophic interactions and balance.

#### GHG reduction synthetic ecosystems

Coastal ecosystems (e.g., mangroves and salt marshes) are significant sources of methane (CH_4_) and nitrous oxide (N_2_O), making them important to manipulate microbe-microbe and host-microbe interactions for mitigating GHG emissions.^66^ These systems function as platforms to decode the electron transfer mechanisms that lead to GHG emissions. For instance, synthetic communities containing nitrogen fixers and N_2_O reducers with key genes *nifH* and *nosZ*, respectively can be inoculated into these ecosystems, utilizing CH_4_ as an energy source for nitrogen fixation while simultaneously mitigating N_2_O emissions.^67^ Constructing synthetic communities of marine bacteria and phytoplankton that interact positively with vitamins and dissolve organic matter (DOM) may also accelerate the production of dimethyl sulfide (DMS), which serves as an anti-GHGs and a key nutrient for marine organisms, while simultaneously promote plant growth.^64^^,^^68^ Additionally, methanotrophs can oxidize CH_4_, with electrons transferred by electroactive bacteria and acetate facilitating electron shuttling, allowing for the conversion of CH_4_ into electricity.^69^ Such microbial CH_4_ oxidation is further enhanced by coupling with CO_2_ reduction through the redox cycling of iron minerals, effectively overcoming energy barriers.^69^ Marine CO_2_ removal through synthetic ecology can be strengthened by geoengineering strategies, such as ocean iron fertilization to stimulate natural phytoplankton blooms and ocean alkalinity enhancement, which uses chemical means to increase the ocean’s CO_2_ absorption capacity, and artificial upwelling, a physical method to bring nutrient-rich deep water to the surface.^70^

#### Carbon sequestration enhancement synthetic ecosystems

Refractory dissolved organic carbon (RDOC), as an important C reservoir produced through a variety of microbial processes by the marine microbial carbon pump (MCP), can effectively slow down global warming.^61^^,^^62^ Therefore, it is crucial to know the interaction within diverse microorganisms for effective RDOC production. Understanding the mechanism of microbial substrate specificity, kinetics of enzymatic oxidation and hydrolysis processes in DOM degradation can help identify conditions that favor the production of more RDOC compounds.^17^ Also, enhancing microbial-driven bicarbonate and carbonate production can raise alkalinity and facilitate the precipitation of RDOC, concurrently increasing the capacity of blue C ecosystems to fix CO_2_.^62^^,^^63^ Synthetic C sequestration-enhancing communities, including autotrophic nitrifiers and heterotrophic auxiliaries (e.g., denitrifiers) through re-fixation of CO_2_ and promotion of necromass and RDOC formation.^63^^,^^64^ Plant growth-promoting microorganisms, especially nitrogen-fixing bacteria converting atmospheric nitrogen into plant-absorbable forms such as ammonia and nitrates, can indirectly sequester C by encouraging plant growth, leading to deep root systems and high C storage in biomass and sediments.^71^ Also, understanding how intensified human activities and global climate change alter interspecies interactions, potentially shifting ecological communities from a stable phase of species coexistence to one marked by compositional instability. These communities may lose their ability to buffer against perturbations, as species may behave synergistically or antagonistically depending on resource availability and habitat conditions.^72^^,^^73^^,^^74^ This knowledge is crucial for improving predictions of climate impacts and ultimately for developing microbial strategies to further mitigate climate warming and C sequestration.

### Limitations of the study

This study proposes the RDVEA framework, yet its implementation faces distinct scientific and technological hurdles. First, a scaling discrepancy exists between laboratory verification and open-ocean deployment, where hydrodynamic dilution and fluid connectivity challenge community cohesion. Also, the predictive power of GSMMs is constrained by incomplete metabolic annotations for the majority of uncultured marine microorganisms. Additionally, while strategies such as “short legacy” traits mitigate risk, the long-term evolutionary stability of synthetic communities against horizontal gene transfer and natural selection in wild environments remains a stochastic variable that current models cannot fully predict. Therefore, these hurdles should be addressed when synthetic ecology theories and approaches are implemented in the future marine ecosystem.

### Conclusion

In conclusion, marine synthetic ecology presents significant potentials for addressing critical marine ecological and environmental challenges. The RDVEA model, combined with advancements in AI, mathematical, and statistical modeling, and multi-omics technologies, provides a structured pathway to develop ecological theories and innovative technologies. In this context, synthetic ecosystems can effectively advance our understanding of multitrophic interactions, mitigating GHG emissions, and enhancing C sequestration in marine ecosystems.

## Acknowledgments

This work is dedicated to the Marine Synthetic Ecology Research Center establishment in May 2024 and supported by the Southern Marine Science and Engineering Guangdong Laboratory (Zhuhai) (SML2020SP004, SML2023SP005, SML2024SP002, and SML2024SP022), the 10.13039/501100001809National Natural Science Foundation of China (42430707 and 92251306), and the Ocean Negative Carbon Emissions (ONCE) Program.

## Author contributions

Conceptualization, R.S. and Z.H.; writing – original draft; R.S.; writing – review and editing, R.S., X.Y., M.N., K.W., H.L., Q.Y. and Z.H.

## Declaration of interests

The authors declare that they have no known competing financial interests or personal relationships that could have appeared to influence the work reported in this article.
